# The relevance of non-human primate and rodent malaria models for humans

**DOI:** 10.1186/1475-2875-10-23

**Published:** 2011-02-02

**Authors:** Jean Langhorne, Pierre Buffet, Mary Galinski, Michael Good, John Harty, Didier Leroy, Maria M Mota, Erica Pasini, Laurent Renia, Eleanor Riley, Monique Stins, Patrick Duffy

**Affiliations:** 1 Division of Parasitology, MRC National Institute for Medical Research, The Ridgeway, London NW7 1AA, UK; 2 Groupe Hospitalier Pitié-Salpêtrière, 47 Boulevard de l'Hôpital, 75651 Paris Cedex 13, France; 3 Yerkes National Primate Research Center, International Center for Malaria Research, Education & Development, Emory University School of Medicine, Emory University, 954 Gatewood Rd, Atlanta, GA 30329, USA; 4 Institute for Glycomics, G26/4.18 Gold Coast Campus, Griffith University, QLD 4222, Australia; 5 Departments of Microbiology and Pathology, and Interdisciplinary Program in Immunology, Carver College of Medicine, University of Iowa, 3-530 Bowen Science Building, 51 Newton Road, Iowa City, IA 52242, USA; 6 Medicines for Malaria Venture, ICC - Block G, 3rd Floor, 20 route de Pré-Bois - PO Box 1826, 1215 Geneva 15, Switzerland; 7 Instituto de Medicina Molecular, Unidade de Malária, Av. Prof. Egas Moniz, 1649-028 Lisboa, Portugal; 8 Biomedical Primate Research Centre (BPRC), Lange Kleiweg, 161, 2288 GJ Rijswijk, The Netherlands; 9 Laboratory of Malaria Immunobiology, Singapore Immunology Network, Agency for Science, Technology and Research (A*STAR) 8A Biomedical Grove, Immunos Building, Biopolis, Singapore 138648 Singapore; 10 Department of Immunology and Infection, Faculty of Infectious and Tropical Diseases, London School of Hygiene and Tropical Medicine, London, UK; 11 RT Johnson Division of NeuroImmunology, Johns Hopkins School of Medicine, 1550 Orleans street, CRB II, Office Room 353/Laboratory Room 362, Baltimore, MD 21231, USA; 12 Laboratory of Malaria Immunology and Vaccinology, NIAID, NIH, 5640 Fishers Lane, Rockville MD 20852, USA

## Abstract

At the 2010 Keystone Symposium on "Malaria: new approaches to understanding Host-Parasite interactions", an extra scientific session to discuss animal models in malaria research was convened at the request of participants. This was prompted by the concern of investigators that skepticism in the malaria community about the use and relevance of animal models, particularly rodent models of severe malaria, has impacted on funding decisions and publication of research using animal models. Several speakers took the opportunity to demonstrate the similarities between findings in rodent models and human severe disease, as well as points of difference. The variety of malaria presentations in the different experimental models parallels the wide diversity of human malaria disease and, therefore, might be viewed as a strength. Many of the key features of human malaria can be replicated in a variety of nonhuman primate models, which are very under-utilized. The importance of animal models in the discovery of new anti-malarial drugs was emphasized. The major conclusions of the session were that experimental and human studies should be more closely linked so that they inform each other, and that there should be wider access to relevant clinical material.

## Background

The 2010 Keystone symposium on malaria (Malaria: New Approaches to Understanding Host-Parasite Interactions), convened leading experts in malaria immunology and pathogenesis to focus on new approaches for understanding host-parasite interactions. Many of the participating scientists conduct their research using rodent or non-human primate experimental models, which have been long-standing tools for malaria immunology and pathogenesis research, basic discovery, drug testing and vaccine development. The relevance of experimental rodent malaria models has recently been a contentious issue in the research community [1-6], and individual participants expressed concern that scepticism about the usefulness of these and other models adversely impacts funding, publishing, critical training opportunities, and the advancement of research. Based on the scientific and practical importance of the topic, the assembled scientists convened a special session to discuss their views on the importance of model systems as tools for understanding human malaria.

## Discussion

Although the major focus of the ensuing discussion was animal models, Nick Anstey (Menzies School of Public Health, Darwin) pointed out that researchers have faced similar challenges when seeking support to investigate human malaria, which often entails studies of association that cannot conclude causality. For this reason, observational research on humans who naturally acquire malaria is sometimes criticized as inconclusive, with the consequence that funding and publication are impeded in this area. Despite these criticisms, studies of malaria in humans are clearly desirable, but many limitations, such as the lack of access to relevant organs and tissue samples, and the inability to manipulate the immune response for mechanistic studies, mandate additional approaches, where animal models may be most appropriate. Furthermore, all meaningful studies on human malaria require appropriately documented samples, which are not always readily available to the global research community.

An example was given by Monique Stins (Johns Hopkins University, Baltimore) who has used an *in vitro *system to identify molecular host-parasite interactions, and would like to confirm these findings using human samples, but, like some others, has been unable to obtain this material thus far. Animal models, therefore, have been the only alternative to test her hypotheses. A consensus of the attendees was the need for funding to establish repositories of human samples in conjunction with carefully collected clinical data, as these would offer invaluable resources to confirm hypotheses in the human disease. This lack of access to human samples often precludes the opportunity to validate findings in existing animal models, thus their relevance remains unproven. Closer collaborations between scientists performing human studies and those performing animal studies are needed to find parallels and differences between these research approaches, to identify which models best approximate human infection and disease.

In certain research areas, animal models are clearly indispensible and are proven tools for discovery. In particular, many of the anti-malarial drugs currently in use emerged from small molecules, whose potencies were assessed in animal models [7]. Historically, drugs were tested for efficacy against different stages of development of rodent parasites, such as *Plasmodium berghei*, to identify those drugs that should proceed to testing against human malaria parasites. Didier Leroy (Medicines for Malaria Venture, Geneva) highlighted recent advances in which the human malaria parasite *Plasmodium falciparum *has been propagated in Severe Combined Immunodeficiency mice receiving continuous injection of human erythrocytes-in other words, an animal model that incorporates the human parasite [8]. This new approach offers the opportunity to assess drug efficacy and pharmacokinetics in an *in vivo *setting against the true parasite target during its blood stage development.

Concerns about the applicability of animal models have been voiced particularly in the areas of disease pathogenesis, malaria immunity and vaccine testing. A generally perceived difficulty is the heterogeneity of malaria presentation in the various mouse-parasite combinations. The outcomes of infection, as well as the nature of the immune responses involved in the development of disease or the acquisition of immunity, can differ widely. This has led to questions about which if any of the mouse models can be extrapolated to understand human disease. Maria Mota (Instituto de Medicina Molecular, Lisbon) emphasized that the spectrum of malaria in the different models may reflect the diversity of human disease or immunity, and thus should be considered a strength rather than a limitation [9-13]. In this view, scientists should use the most appropriate model for their particular research question rather than expect one model to represent all facets of human malaria.

Cerebral malaria in mice is one of the areas of greatest controversy. *Plasmodium berghei *ANKA strain causes accumulations of inflammatory cells and neurological symptoms leading to death in C57BL/6 mice. One of the major differences between human cerebral malaria and the *P. berghei *ANKA models is thought to be the extent of parasite sequestration in the brain. In rodents with cerebral malaria this has always been thought to be low, whereas parasite sequestration in the brain is a characteristic feature of human cerebral malaria. Numerous cells, cytokines and chemokines have all been found to play a role in cerebral malaria in the rodent models, but their importance in human cerebral malaria is still debated. To counter these criticisms, some scientists at the meeting presented evidence of similarities between the animal models and human disease. Laurent Renia (A*, Singapore) itemized numerous features of both human and rodent cerebral malaria, and concluded that the two had significant consistencies. Specific strains of parasites, for example *P. berghei *ANKA, do sequester in the rodent brain, however there is a need to define carefully the parasite used and how this parasite is produced and infection initiated. Eleanor Riley showed histological images (kindly provided by Kevin Couper, LSHTM, London) documenting accumulations of sequestered *P. berghei *ANKA parasites in brains of infected mice. Importantly it was pointed out that the parasite burden in the human brain during cerebral malaria can vary widely between individuals, and in some cases can be relatively low despite the severe syndrome.

John Harty (University of Iowa) noted that much of what is known at a basic level of immunology emerged from studies of infectious diseases in mice, and that most of this knowledge is applicable to humans despite minor differences in pathways, receptors and cytokines/chemokines. Eleanor Riley pointed out the concordance between immune regulatory mechanisms in humans and mice, and cited an example of IL-10 production from IFNγ-CD4 T cells being first observed in humans recovering from *P. falciparum *malaria, and their regulatory role subsequently being verified in a mouse model. On a practical level, the mouse malaria models may have not always predicted the vaccines that would succeed in humans, however they have always reliably predicted the vaccine failures, thus preventing further development of ineffective vaccines. One possible approach for improving rodent models for dissecting human immune responses may be the use of humanized mice and/or chimaeric rodent parasites carrying an introduced gene from human malaria parasites. However in the view of several scientists attending the symposium their advantages over standard rodent models was not yet clear, aside from the possibility that human hepatocytes and erythrocytes in rodents will allow the growth of human malaria parasites.

Primate malarias were cited as alternative models for humans and examples of useful models were reviewed. Mary Galinski (Yerkes National Primate Research Center, Emory University, Atlanta) discussed the importance of several non-human primate malaria models. She emphasized that many of the key human malaria syndromes and research problems have excellent primate models that are underutilized, and that increased resources and collaborations with investigators asking clinically relevant questions could resolve this problem. Erica Pasini (BPRC, The Netherlands) described *Plasmodium coatneyi *in the rhesus monkey as a model of parasitized red cell sequestration, while *Plasmodium knowlesi *in non-human primates can serve as model for *P. knowlesi *human infections in South East Asia, some of which are characterized by extreme severity [14,15]. Also *Plasmodium cynomolgi *is closely related and mimics the unique biology and pathogenesis of *Plasmodium vivax*. This species offers opportunities to study the dormant relapsing parasite forms called hypnozoites, and the unique infected red blood cell features of *P. vivax*, to help identify drug and vaccine targets. *Plasmodium knowlesi*, also found in humans, has traditionally provided knowledge on merozoite invasion and antigenic variation. Galinski highlighted the critical need for sequencing the genomes of these primate malaria parasites, with closure to enable gene expression studies, and the possibility that these valuable models might be lost unless young scientists are supported to gain expertise utilizing these non-human primate resources.

Over-interpretation of data, particularly with respect to therapeutic intervention, has prompted further skepticism toward experimental models. In mouse models, inhibiting or blocking cells, cytokines and immune pathways, often promoted as potential therapies, are carried out before onset of symptoms. Whilst these are valid approaches to dissect mechanisms of pathogenesis, they are not useful guides for treating human severe disease, where intervention usually is initiated only after onset of symptoms. Thus many adjunctive therapies for severe malaria that have been effective in mice have failed in human studies. Treatments of infected animals therefore need to parallel more appropriately the manner in which treatment is given to humans, and should be assessed for efficacy when started after the appearance of clinical signs. In addition, delivering adjunctive treatments in conjunction with other standard anti-malarial therapies, such as quinine, would better model therapy delivered to humans.

The consensus of the Keystone attendees coalesced around *the need to reconcile the opposing views *of "no animal model replicates human malaria", and "animal models are always useful, or are the only approach possible to dissect mechanisms of immunity or immunopathology", in order to move research forward. Pierre Buffet (Paris) suggested that models should be selected that best reflect the particular human syndrome or response, and that similarities, differences and limitations of each model should always be described for each proposed application. Immune mechanisms and processes are more likely to have relevance for human malaria if they are verified in different experimental systems, with consistencies between several models, from mouse to primate. Proposed therapies should be examined when given after a syndrome has developed, and efforts should be made to examine these therapies in conjunction with other interventions typically given to treat humans with malaria.

## Conclusions

Experimental and human studies must be closely linked so that they each inform the other. To this end, bringing together scientists who study human malaria with those who study experimental models to compare and contrast the details of each might be especially fruitful. These discussions can identify the areas where the models are currently known to be useful, and the areas for further study that might identify where the models are or are not appropriate.

## Authors' contributions

All authors contributed to the discussion and writing of the manuscript, and have read and approved the final manuscript.
